# Locum doctor use in English general practice: analysis of routinely collected workforce data 2017–2020

**DOI:** 10.3399/BJGP.2021.0311

**Published:** 2022-01-11

**Authors:** Christos Grigoroglou, Kieran Walshe, Evangelos Kontopantelis, Jane Ferguson, Gemma Stringer, Darren M Ashcroft, Thomas Allen

**Affiliations:** Manchester Centre for Health Economics, University of Manchester, Manchester, UK.; Alliance Manchester Business School, Institute for Health Policy and Organisation, University of Manchester, Manchester, UK.; NIHR School for Primary Care Research, Centre for Primary Care, University of Manchester, Manchester, UK; Division of Informatics, Imaging and Data Science, University of Manchester, Manchester, UK.; Alliance Manchester Business School, Institute for Health Policy and Organisation, University of Manchester, Manchester, UK.; Alliance Manchester Business School, Institute for Health Policy and Organisation, University of Manchester, Manchester, UK.; NIHR School for Primary Care Research, Centre for Primary Care, University of Manchester, Manchester, UK; NIHR Greater Manchester Patient Safety Translational Research Centre, University of Manchester, Manchester, UK.; Manchester Centre for Health Economics, University of Manchester, Manchester, UK; Danish Centre for Health Economics, University of Southern Denmark, Denmark.

**Keywords:** employment, general practice, GP locums, health workforce, regional variation, statistics and numerical data

## Abstract

**Background:**

Numbers of GP locums in the NHS have grown in recent years, yet evidence on the scale and scope of the locum workforce in general practice is sparse.

**Aim:**

To identify characteristics, geographical patterns, and drivers of GP locum use.

**Design and setting:**

Observational study of routine data from general practices in England.

**Method:**

Descriptive analyses of national GP workforce data between December 2017 and September 2020 were conducted to determine the volume and geographical distribution of locum use and examine the characteristics of locums compared with other GP types. Locum full-time equivalent (FTE) was modelled using negative binomial regression and estimated incidence rate ratios (IRRs) for associations between outcome and characteristics of practices and population.

**Results:**

In December 2019, total locum FTE was 1217.9 compared with 33 996.6 for total GP FTE. Locums represented 3.3% of total GP FTE, which was fewer than other GP types. Median locum age was 42 years (interquartile range [IQR] 36 to 51) FTE and the majority were UK qualified (660 of 1034 [63.8%] total locum FTE), were male (642.6 of 1178.9 [54.5%] total locum FTE), and had long-term employment (834.1 of 1127.9 [74.0%]) total locum FTE. Rurality (IRR 1.250 [95% CI = 1.095 to 1.428]), inadequate Care Quality Commission ratings (IRR 2.108 [95% CI = 1.370 to 3.246), and single-handed practice (IRR 4.611 [95% CI = 4.101 to 5.184) were strong predictors of locum use. There was substantial variation in locum use between regions.

**Conclusion:**

GP locum use remained stable over time. Compared with other GPs, locums were younger male GPs, a substantial percentage of whom did not qualify in the UK, and those who served underperforming practices in rural areas. This is likely to reflect recruitment or high turnover challenges in these practices/areas and can provide a greater understanding of general practice workforce challenges in England.

## INTRODUCTION

Access to primary healthcare services is a core dimension for any high-quality healthcare system^1^^,^^2^ and higher availability of primary care services has been associated with lower all-cause mortality, lower hospital admission rates, and lower healthcare costs.^3^ However, general practice faces multiple challenges to reduce service access inequalities,^4^^,^^5^ such as insufficient staff and capacity to meet rising patient need and complexity that have a direct impact on quality of patient care and staff experience.^6^ A flexible GP workforce can help combat these challenges, but there is little research on the scale and scope of temporary GP (usually referred to as locums) use in the UK.

In recent years the NHS has suffered from insufficient long-term workforce planning, prolonged shortfalls in funding, and a high number of doctors leaving the profession early,^7^ contributing to the current workforce crisis. The GP primary care workforce has been severely affected by staffing problems,^8^ compounded by excessive workload and burnout during the COVID-19 pandemic in 2020. Locum GPs are defined as doctors who provide cover for permanent staff including maternity/paternity leave, sick leave, annual leave, suspended doctors, or vacancies. Locum GP contracts are arranged at the practice level to tackle short-term staff flexibility and to fill service gaps in remote, small rural areas and in single-handed practices, where locum working may be the only way to obtain cover for sickness or annual leave. In the NHS, trends towards non-standard forms of work^9^ have led to increases in the numbers of locum GPs, with an increase of 9.7% in GP locum full-time equivalent (FTE) use between June 2017 and June 2019, with this figure accounting for 3.8% of total GP FTE use in June 2019.^10^

However, NHS organisations need more detailed information to effectively use their workforce. This study aimed to quantify general practice GP locum use in England as an aggregate and by clinical commissioning group (CCG) by age group, country of qualification, and sex, for the entire GP locum workforce of England; and make comparisons with other types of GPs. The study also aimed to examine which practice and population characteristics explained variability in locum use at the general practice level for the entire primary care population of England.

**Table:** How this fits in

Prior research on the extent of GP locum use in general practice and the composition of the GP locum workforce is sparse. The availability of new data from general practice allows an opportunity to generate new knowledge and to add to the understanding of the current GP workforce composition. Results of the present study suggest that GP locum use has remained stable over time and comparisons of GP locums with other types of GPs show that locums are mostly younger male doctors, of whom a large proportion have qualified elsewhere other than the UK, and work in underperforming practices. Substantial regional variation in GP locum use across England indicate differencesin workforce planning, recruitment, and retention. This research provides a useful approach to measure the extent of locum use in primary care and can aid workforce planning by identifying areas of increased recruitment, areas with high GP turnover, and also the drivers behind variation in locum use in English primary care.

## METHOD

### Data sources

Several data sources were accessed to extract individual-level information on FTE working hours (1 FTE = 37.5 hours/week), type of GP: locum, partner, registrar, junior, retainer, and salaried; type of locum GP: long-term or infrequent locum; and GP characteristics of age, sex, country of qualification. General practice-level information was extracted on population age and sex, quality of care, morbidity burden, patient satisfaction, rurality, deprivation, single-handed practices, and healthcare regulators’ rating for each general practice in England. Definitions and sources for all data are provided in Supplementary Tables S1–3.

Practitioner-level information from NHS Digital was extracted from practices on the last day of each reporting quarter, with 12 quarters available between 31 December 2017 to 30 September 2020. The time-period window was restricted as there were differences in methodology used by practices to report locum data before December 2017. For the period of analysis, some practices did not provide valid or complete records, and this resulted in some data being recorded as missing or estimated. Even though these records were excluded from the analyses, coverage was very high with approximately 95% of all practices providing valid workforce data in December 2019. The FTE for locum GPs was derived as an average of the total number of hours worked in each month over the reporting quarter.^11^

Information on achievement indicators for all long-term conditions in the UK’s Quality and Outcomes Framework (QOF) was obtained from NHS Digital.^12^ This information was used to calculate morbidity burden and performance for each general practice in the dataset. The QOF is a national pay-for-performance scheme in primary care, which was introduced in 2004 with the aim to improve quality of care, and linked financial awards to performance on achievement indicators. Detailed information on the conditions, their indicators, and the methodology used to calculate these measures are provided in Supplementary Table S2. Lower-Layer Super Output Area (LSOA)-level deprivation, as measured by the Index of Multiple Deprivation (IMD) 2019,^13^ was available for all LSOAs (geographically defined neighbourhoods of 1500 people on average) and LSOA deprivation scores were assigned to practices based on the practice’s postcode.

The IMD is a relative measure of deprivation for all 32 844 LSOAs in England where each LSOA is assigned a score on a continuous scale, that is, 0–100, and a higher score corresponds to greater deprivation. Data on patient satisfaction were extracted for all practices from the General Practice Patient Survey,^14^ data on rural/urban classification were based on practices’ location,^15^ and practice overall inspection ratings were extracted from the Care Quality Commission (CQC).^6^ Data were publicly available and did not require ethical board review.

### Statistical analysis

Total locum FTE was plotted against total FTE for all types of GPs over time for the whole of England. Violin plots were used to compare GP age and FTE distribution of locums and other GP types, and by sex. A practice’s use of locums is defined as locum FTE as a proportion of total GP FTE. Spatial maps were used to visualise geographical variation in mean locum use at the CCG level for 2019.

To model practice locum FTE, mean-dispersion negative binomial models were used with robust standard errors, and with fixed-effects predictors for region (as categorical, to account for between-region variations) and time (as continuous, to account for time trends). The natural logarithm of total GP FTE was used as offset. Several practice characteristics were controlled for in all models: deprivation, practice CQC ratings, proportion of practice’s female population, proportion of practice’s patients aged ≥65 years, single-handed practices, rurality, QOF performance, QOF morbidity burden, patient satisfaction, and practice workload defined as list size over total GP FTE. One set of negative binomial regression models was used to investigate the relationship between locum use and practice and population characteristics over time (2018 to 2019) and one set of models to investigate the relationship cross-sectionally (2019).

Stata (version 16.1) was used for the principal data cleaning, management, and analyses. For the two primary sets of analyses, the *nbreg* command with the exposure option and the incidence rate ratio (IRR) specification was used. Practices with <1000 patients were omitted from the regression analyses because these practices are opening, closing, or serving specific populations.

## RESULTS

Over time, aggregate reported locum use in England varied from 3.15% (1045.8 of 33 133.4 total GP FTE), in December 2017, to 3.08% (1040.9 OF 33 801.1 total GP FTE), in December 2018, to 3.58% (1217.9 of 33 996.6 total GP FTE), in December 2019, to 3.31% (1157 of 34 850.6 total GP FTE) in September 2020 (Figure 1a). The proportion of practices that reported at least some locum use varied from 37.4% and 40.8% in 2018 and 2019, respectively. Most locums (834.1 of 1127.9 FTE [74%]) worked in long-term positions compared with infrequent locums (26%) (Figure 1b). Long-term locum use remained stable over the study period, though there was a substantial 47% reduction in infrequent locum use between the last quarter of 2019 and the first quarter of 2020, indicating the impact of the COVID-19 pandemic.

Violin plots depicting the FTE distribution of locums and other types of GPs are presented in Figure 2a. Median locum FTE in December 2019 was 0.09 FTE (0.7 sessions in a practice where 1 FTE = 8 sessions) and a similar distribution was observed in FTE for both male and female locums in contrast with other GP types (for example, GP partners/salaried GPs) where a large variation in the distribution of FTE between sexes was observed.

**Figure 1a.**
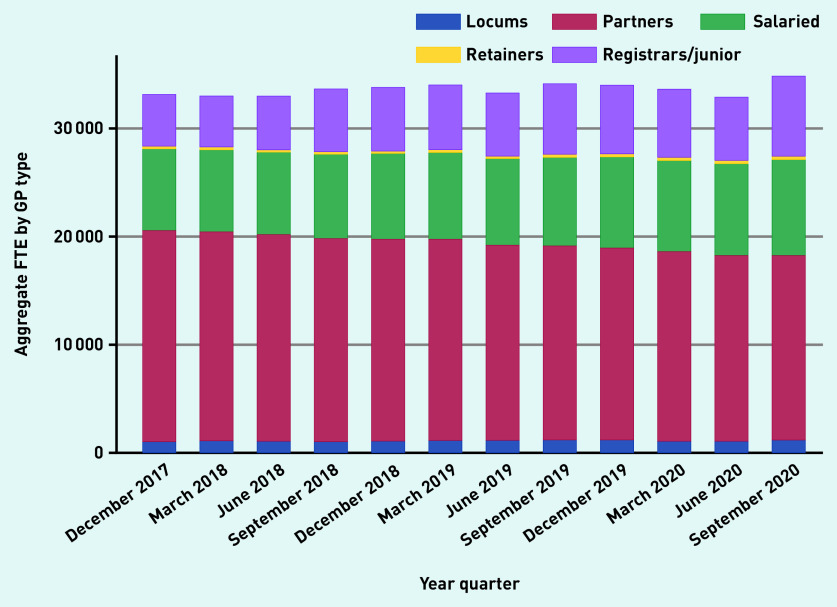
*Variation in FTE by GP type over time, December 2017 to September 2020.* *FTE = full-time equivalent.*

**Figure 1b.**
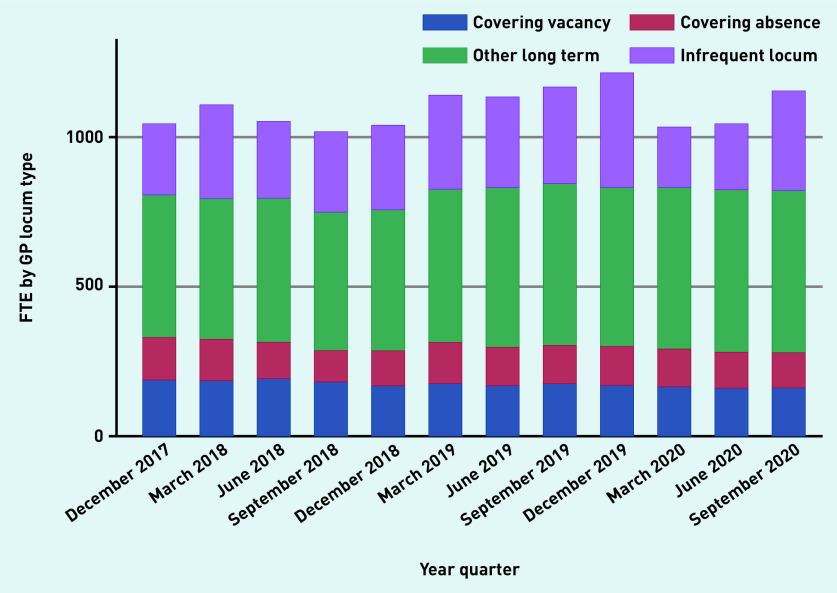
*Variation in FTE by locum type over time, December 2017 to September 2020.* *FTE = full-time equivalent.*

**Figure 2a.**
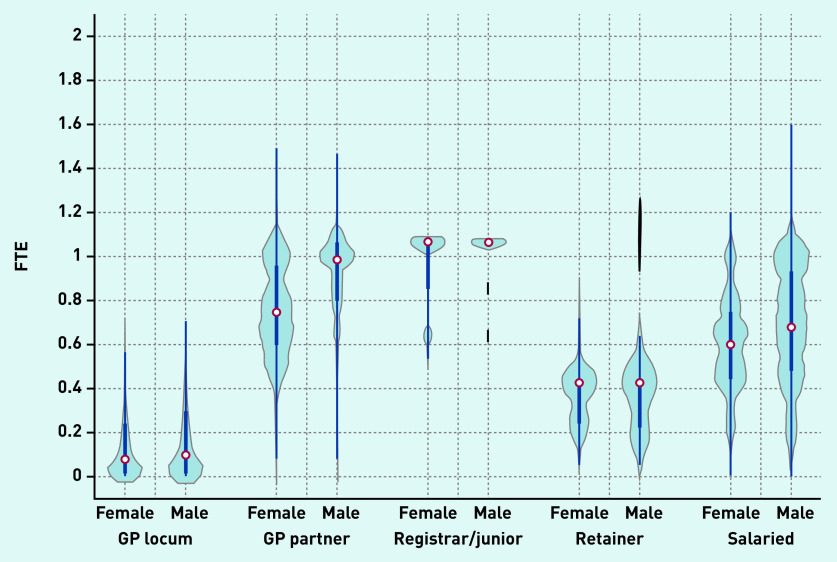
***Full-time equivalent (FTE) distribution of GPs in December 2019 by type and sex.*** *^a^* *^a^* ***The thick blue line represents the interquartile range (IQR) and the thin line represents the rest of the distribution with upper/lower adjacent values. The red dot represents the median of the data. The distribution shape of the data is based on a kernel density estimation where wider sections of the plot represent a higher chance that members of the population of interest will take on a given value and where the thinner sections represent lower chance.***

Locum workforce age characteristics are presented in Figure 2b and sex and country of qualification characteristics are presented in Figure 3a and 3b. In December 2019, the age distribution of locums shared similar characteristics with the age distribution of both male and female salaried GPs and with the age distribution of female GP retainers. The median age of GP locums was 42 years (IQR 35 to 55). Female locums had a median age of 41 years (IQR 35 to 48) and male locums had a median age of 44 (IQR 37 to 55) (Figure 2b). The median age for GP partners, junior doctors, GP retainers, and salaried GPs was 49, 31, 42, and 39 years respectively.

**Figure 2b.**
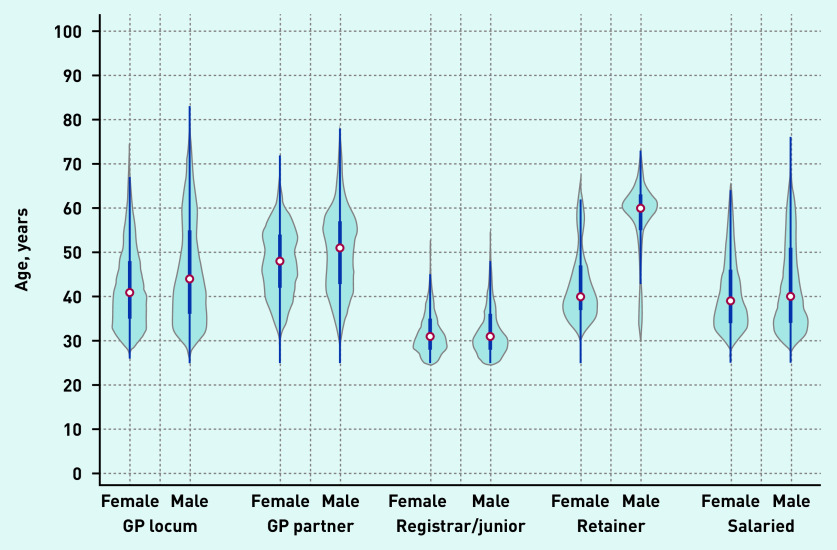
***Age distribution of GPs in December 2019 by type and sex.*** *^a^* *^a^* ***The thick blue line represents the interquartile range (IQR) and the thin line represents the rest of the distribution with upper/lower adjacent values. The red dot represents the median of the data. The distribution shape of the data is based on a kernel density estimation where wider sections of the plot represent a higher chance that members of the population of interest will take on a given value and where the thinner sections represent lower chance.***

**Figure 3a.**
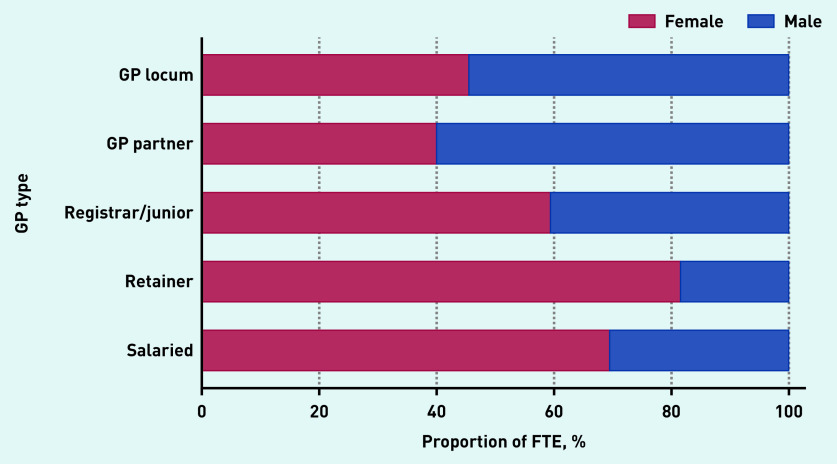
*Sex breakdown by GP type, December 2019. FTE = full-time equivalent.*

**Figure 3b.**
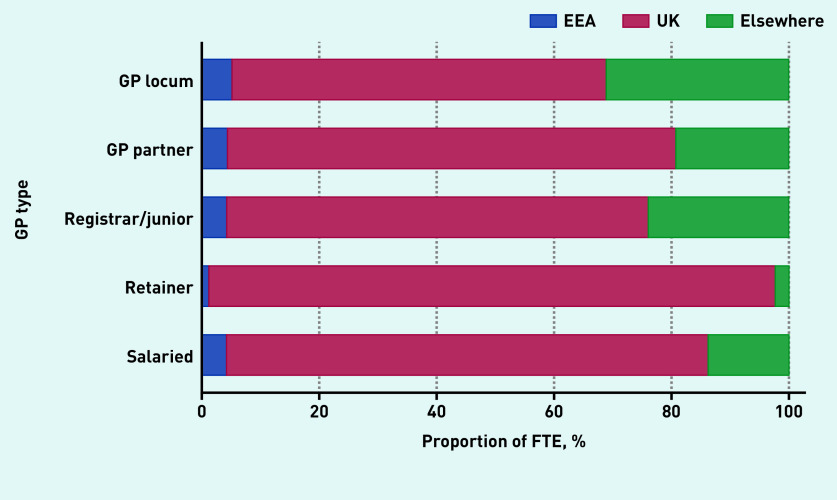
*Country of qualification breakdown by GP type, December 2019.* *EEA = European Economic Area.*

Male locums accounted for 54.5% of total locum FTE, which was similar to GP partners, who were mostly male (60%). In contrast, registrars/junior doctors (40.6%), GP retainers (18.5%), and salaried GPs (30.5%) were mostly female (Figure 3a).

**Figure 4.**
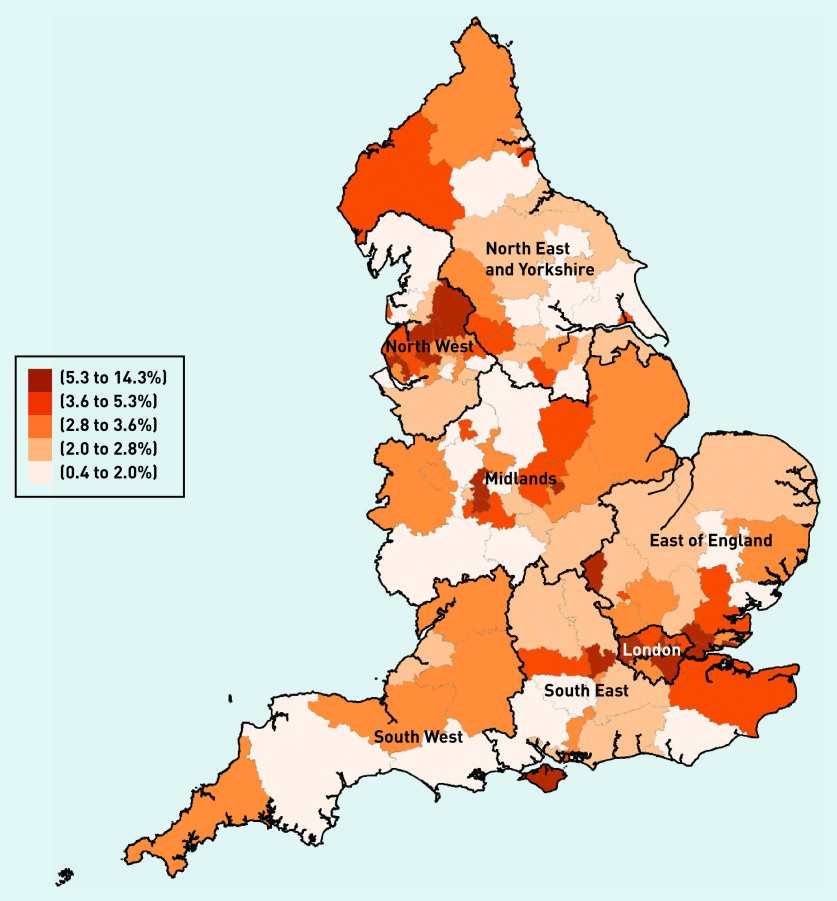
***Geographical distribution of locum use in 2019.*** *^a^* *^a^* ***Locum use is defined as mean locum FTE as a proportion (%) of total GP FTE. Population is divided into quintiles depending on levels of locum use. A higher level of the measure in the key represents higher locum use in the locality. FTE = full-time equivalent.***

In terms of country of qualification, most locums obtained their degrees in the UK (63.8%), though this proportion was smaller compared with other types of GPs (82% for salaried GPs and 76% for partner GPs) (Figure 3b). Variability in mean locum use at the CCG level across regions in 2019 is presented in Figure 4. In the Figure 4, darker-shaded areas indicate higher locum use and lighter-shaded areas indicate lower locum use. Locum use varied substantially between regions (from 0.4% to 13.7%) with locum use accounting for 2.5% of total GP FTE in the North East and 7.4% in London.

Descriptive statistics on locum FTE, population size estimates, number of practices, census information, deprivation (IMD), and QOF population achievement for all English regions in 2019/2020 are reported in Table 1.

**Table 1.** Descriptive statistics in 2019 by region

**Practice-level characteristics**	**Region**							
	**England**	**North East and Yorkshire**	**South West**	**East of England**	**North West**	**South East**	**Midlands**	**London**
Locum GP FTE, yearly mean (95% CI)	192.1 (125.0 to 259.2)	136.9 (118.9 to 154.8)	137.8 (130.2 to 145.3)	155.1 (147.1 to 163.2)	165.1 (159.6 to 170.7)	165.6 (152.5 to 178.6)	254.8 (234.3 to 275.4)	329.5 (323.5 to 335.5)
Total GP FTE, yearly mean (95% CI)	4564.1 (1967.4 to 6639.6)	5457.9 (2754.6 to 8154.1)	3628.0 (1849.7 to 5396.3)	3718.0 (1911.5 to 5497.5)	4408.0 (1805.9 to 6983.0)	4869.0 (2238.4 to 7471.5)	6317.2 (2937.3 to 9699.3)	4471.7 (1696.9 to 7254.1)
Locum use (%)^a^	4.2	2.5	3.8	4.2	3.7	3.4	4.0	7.4
General practice population, *n*	57 653 853	8 788 992	5 477 907	6 692 664	7 057 650	9 178 676	10 780 976	9 676 986
Practices, *n*	6422	991	539	655	951	856	1268	1162
Single-handed practices, *n*	685	93	8	75	146	68	157	138
Practice list size, *n* median (IQR)	7522.0 (4692.0 to 11 124.0)	7708.8 (4913.3 to 10 884.5)	8760.8 (5996.8 to 12 481.5)	8997.0 (6065.0 to 12 868.5)	6410.3 (4212.3 to 9315.8)	9845.3 (6367.4 to 13 180.0)	7333.5 (4591.8 to 10 829.6)	7311.5 (4858.0 to 10 554.0)
IMD 2019,^b^ median (IQR)	21.9 (12.5 to 35.5)	29.3 (15.7 to 46.8)	18.2 (11.4 to 26.0)	16.6 (9.2 to 24.5)	32.5 (17.3 to 52.8)	14.2 (7.6 to 22.5)	25.0 (14.3 to 39.8)	22.1 (13.6 to 30.7)
Practice female population, median (IQR) (%)	3778.0 (2315.0 to 5611.0) (50.0)	3851.0 (2428.5 to 5437.8) (50.0)	4392.5 (3035.8 to 6365.5) (50.1)	4529.0 (3018.3 to 6510.8) (50.3)	3165.8 (2088.0 to 4686.0) (49.4)	4956.4 (3228.6 to 6670.6) (50.3)	3639.3 (2250.3 to 5441.9) (49.6)	3617.3 (2380.3 to 5248.5) (49.5)
**QOF data**								
Population achievement, % median (IQR)	82.2 (79.7 to 84.4)	83.1 (80.8 to 85.0)	82.6 (80.1 to 84.3)	82.5 (80.1 to 84.6)	82.6 (80.2 to 84.7)	81.8 (79.4 to 83.9)	82.5 (79.8 to 84.6)	80.6 (78.0 to 83.2)
Morbidity burden, % median (IQR)	67.0 (55.0 to 77.3)	75.7 (67.1 to 83.2)	73.2 (64.9 to 80.4)	64.8 (56.3 to 73.0)	74.2 (65.5 to 82.2)	64.2 (56.5 to 73.5)	71.0 (62.4 to 79.4)	50.6 (43.2 to 57.2)
Rural, %	15.4	17.2	32.5	27.1	5.3	21.4	18.0	0.1

a *Locum use is defined as mean locum FTE as a proportion (%) of total GP FTE,*

b *IMD measures the deprivation of the area in which a practice is located. A higher value indicates greater deprivation. The IMD values are on a scale of 0 to 100. CI = confidence interval. FTE = full-time equivalent. IMD = Index of Multiple Deprivation. IQR = interquartile range. QOF = Quality and Outcomes Framework.*

Supplementary Table S1 shows the 10 CCGs with the highest use of locums and 10 CCGs with the lowest use of locums in 2019 and their characteristics.

### Regression analyses

Partial results from the over-time and cross-section regression models (A and B respectively) are reported in Table 2 and the full regression results are reported in Supplementary Table S3. After adjusting for practice and population characteristics, large variability in locum FTE between regions persisted. Using the Midlands as the reference category, practices in London had the highest locum FTE (IRR 1.369, 95% CI = 1.180 to 1.588), and practices in the North East and Yorkshire had the lowest locum FTE (IRR 0.711, 95% CI = 0.626 to 0.843) (Supplementary Table S3).

**Table 2.** Regression analyses results from negative binomial regression for locum use at general practice level, Model A: over time (2018–2019), Model B: cross-sectionally (2019)^a^

**Characteristic**	**Model A IRR (95% CI)**	**Standard error**	***P*-value**	**Model B IRR (95% CI)**	**Standard error**	***P*-value**
Rurality (0 = urban, 1 = rural)	1.250 (1.095 to 1.428)	0.085	<0.001	1.300 (1.085 to 1.559)	0.120	<0.004
IMD 2019	1.002 (0.999 to 1.006)	0.002	<0.096	1.005 (1.000 to 1.009)	0.002	<0.046
QOF practice performance	1.005 (0.991 to 1.017)	0.007	<0.479	1.009 (0.991 to 1.026)	0.009	<0.298
Single-handed practice	4.611 (4.101 to 5.184)	0.276	<0.001	4.618 (3.928 to 5.428)	0.381	<0.001
QOF morbidity burden	1.384 (0.963 to 1.991)	0.257	<0.079	1.255 (0.801 to 1.996)	0.287	<0.320
Percentage of female population	0.967 (0.959 to 0.981)	0.006	<0.001	0.970 (0.946 to 0.994)	0.012	<0.015
Proportion of practice population aged ≥65 years	0.970 (0.950 to 0.984)	0.009	<0.001	0.971 (0.958 to 0.988)	0.007	<0.001
Practice workload (total GP FTE/list size)	1.001 (1.001 to 1.002)	0.001	<0.001	1.001 (1.001 to 1.002)	0.001	<0.003
**CQC ratings (reference group is outstanding services)**	Reference group			Reference group		
Inadequate	2.108 (1.370 to 3.246)	0.464	<0.001	2.687 (1.451 to 4.974)	0.844	<0.001
Requires improvement	1.229 (0.949 to 1.592)	0.163	<0.118	1.198 (0.822 to 1.744)	0.229	<0.346
Good	1.343 (1.103 to 1.637)	0.136	<0.003	1.267 (0.947 to 1.696)	0.188	<0.111
**Year (reference year is 2018)**	Reference year			—		
2019	1.055 (0.970 to 1.148)	0.045	<0.210	—		
Constant	0.041 (0.011 to 0.142)	0.026	<0.001	0.020 (0.004 to 0.111)	0.018	<0.001

a *Locum use is defined as practice aggregate FTE of locum doctors. QOF performance is measured as % achievement of the population across all QOF indicators. Coefficients can be interpreted as percentage change, for example, adjusted locum use in London was 0.45% lower than the East of England (Model A). CI = confidence interval. CQC = Care Quality Commission. FTE = full-time equivalent. IMD = Index of Multiple Deprivation. IRR = incidence rate ratio. QOF = Quality and Outcomes Framework.*

CQC ratings appeared to be a strong predictor of locum FTE, where practices rated as having inadequate (IRR 2.108, 95% CI = 1.370 to 3.246) and good services (IRR 1.343, 95% CI = 1.103 to 1.637) had higher locum FTE than practices that were rated as having outstanding services (Table 2). Single-handed practices had substantially higher locum FTE (IRR 4.611, 95% CI = 4.101 to 5.184) compared with group practices. For practices in rural locations, locum FTE was 25% higher than for practices located in urban areas (IRR 1.250, 95% CI = 1.095 to 1.428). Practices with a higher proportion of female population had 3.3% lower locum FTE (IRR 0.967, 95% CI = 0.959 to 0.981) than practices that had a higher proportion of male population. A larger patient population in the ≥65 years age group was associated with 3% lower locum FTE (IRR 0.970, 95% CI = 0.950 to 0.984) (Table 2).

Finally, patient satisfaction was very weakly associated with locum FTE while deprivation, QOF quality of care, practice performance, QOF morbidity burden, and practice workload did not appear to have any discerning effect on practice locum FTE.

## DISCUSSION

### Summary

This study describes a methodological approach to capture and monitor the scale and scope of the GP locum workforce in English primary care. The presented findings suggest that between December 2017 and September 2020 the proportion of GP locum work in the NHS has remained stable, despite widespread perceptions that numbers of locum GPs have risen.^16^ Regarding regional variation and the characteristics of locums, the authors describe the intensity of locum use in general practice and how this varies across regions, as well as important information about the composition of the GP locum workforce. This study identified substantial geographical variation in locum use between and within regions suggesting differences in the distribution of locums in England. Comparisons of locums with other GP types showed that locums were more mobile, younger males of whom most had qualified in the UK, though a large percentage had qualified elsewhere. Most locums were employed in long-term positions and on average they did very few sessions. The regression analyses results showed that practice characteristics such as rurality, CQC ratings, and whether the practice was single-handed were stronger predictors of higher locum FTE than population characteristics.

Locum GPs have an important role in the delivery of primary care services, particularly in the delivery of out-of-hours care and in helping to address short-term workforce shortages. Despite expectations that locum GP numbers are rising, the study found that locum use in primary care has remained stable over time, though the use of locums seems to vary substantially across different practice types and areas of the country.

### Strengths and limitations

This analysis was conducted at the population level and quantified and examined the scale and characteristics of the GP locum workforce compared with other types of GPs for the first time across general practices in England. The study explored whether variation in practice and population characteristics explain variability in locum FTE to account for various health needs across different practice populations. The study has national scope and comprehensive coverage of the primary care population (95% of all general practices).

Publicly available routinely collected data from NHS Digital were used in this study. However, other databases on workforce report different estimates on the numbers of locum GPs. The General Medical Council (GMC) register and the National Association of Sessional GPs (NASGP) estimate approximately 17 000 to 18 000 GPs with a locum licence in England in 2017,^16^^,^^17^ while the NHS Digital data report showed there were only 5040 employed locum GPs in December 2017.^10^ There may be several reasons why these differences exist. First, NHS Digital data show a picture of the actual GP workforce at each time point rather than the prospective workforce that other databases report. Locum headcounts may overestimate locum use as some locum GPs may also be simultaneously permanently employed.

Second, the GP workforce data collected by NHS Digital have been subjected to changes in data sources and methodology over the years and also include estimates for practices where data are incomplete or have not been submitted. The authors restricted their time period to exclude data before December 2017 when the infrequent locum category was first reported in the collection and excluded estimates for those practices that did not submit valid data.

Third, locum data are believed to be under-reported when compared with other types of GPs, mainly because of the infrequent locum category for which reporting may be lower than long-term locum data.

In September 2020 NHS Digital switched from quarterly to monthly data collections of the GP workforce data; however, the transition to monthly collection led to a decrease in the number of FTE for infrequent locums. For this reason, the data collections were reverted to quarterly to allow practice managers to report infrequent locum data in time.^15^

### Comparison with existing literature

Previous international evidence shows that the numbers of locums continue to rise,^9^^,^^16^^,^^18^ but the present findings suggest that this may not be the case for GPs in England. Previous reports from the GMC and NASGP showed that the proportion of GPs with a locum GP contract had increased from 30% to 39% of all licensed GPs from 2013 to 2016 and was equivalent to approximately 18 000 GPs in 2018.^16^

One recent study examined the geographical variation in the distribution of the GP workforce, including GP locums, across the 13 Health Education England (HEE) regions using data from NHS Digital but did not make specific comparisons between locums and other types of GPs.^19^ The comparisons in the present study can provide a review of the locum workforce at a more granular level that is also particularly relevant to NHS organisations, for example, CCGs. To the authors’ knowledge, no other studies to date have examined contextual factors and their association with locum use in general practices.

### Implications for research and practice

The accurate monitoring of the GP workforce may help policymakers and commissioners to understand current challenges in primary care, including capacity and composition of the GP workforce and inform workforce planning. This can be particularly useful to meet local healthcare needs with sufficient resources for training and deployment of GPs, which will help ensure that the targets set out in the NHS Long Term Plan are met.^20^

For example, this research highlights elevated locum GP employment in practices in rural areas and those with inadequate CQC inspection ratings. These types of practices may face substantial challenges in recruiting and retaining permanent GPs, and it may be hypothesised that relatively high and sustained levels of locum use may be indicators of wider problems affecting recruitment and retention.

Furthermore, the present study lays the foundation for future analysis of other existing routine primary care datasets that contain information on service utilisation and patient outcomes. Additional work is needed to identify whether differences in clinical practice and performance between locum doctors and permanent doctors exist as well as the consequences these may have for patient safety and quality of care. Future work should also aim to identify career intentions of locum GPs and factors that influence their choice to work as a locum. It will be important to understand the implications of these career intentions and what the observed locum workforce characteristics have on future workforce planning. As more data become available, the impact of COVID-19 on the use of the GP locum workforce should be examined.

## Data

The data used in this study are freely available and were obtained from NHS Digital. The authors are happy to share in an organised and cleaned final dataset all publicly available data.
